# Primary care REFerral for EchocaRdiogram (REFER) in heart failure: a diagnostic accuracy study

**DOI:** 10.3399/bjgp16X688393

**Published:** 2016-12-06

**Authors:** Clare J Taylor, Andrea K Roalfe, Rachel Iles, FD Richard Hobbs, The REFER investigators, P Barton, J Deeks, D McCahon, MR Cowie, G Sutton, RC Davis, J Mant, T McDonagh, L Tait

**Affiliations:** Nuffield Department of Primary Care Health Sciences, University of Oxford, Oxford.; Institute of Applied Health Research, University of Birmingham, Birmingham.; Institute of Applied Health Research, University of Birmingham, Birmingham.; Nuffield Department of Primary Care Health Sciences, University of Oxford, Oxford.; Institute of Applied Health Research, University of Birmingham, Birmingham.; Institute of Applied Health Research, University of Birmingham, Birmingham.; Institute of Applied Health Research, University of Birmingham, Birmingham.; Faculty of Medicine, National Heart and Lung Institute, Imperial College London.; Faculty of Medicine, National Heart and Lung Institute, Imperial College London.; Department of Cardiology, Sandwell and West Birmingham Hospitals, Lyndon, West Bromwich.; Department of Public Health and Primary Care, University of Cambridge, Cambridge.; Department of Cardiology, King’s College Hospital, London.; School of Health Sciences, Nottingham.

**Keywords:** clinical decision rule, diagnostic, echocardiography, general practice, heart failure, natriuretic peptide

## Abstract

**Background:**

Symptoms of breathlessness, fatigue, and ankle swelling are common in general practice but deciding which patients are likely to have heart failure is challenging.

**Aim:**

To evaluate the performance of a clinical decision rule (CDR), with or without N-Terminal pro-B type natriuretic peptide (NT-proBNP) assay, for identifying heart failure.

**Design and setting:**

Prospective, observational, diagnostic validation study of patients aged >55 years, presenting with shortness of breath, lethargy, or ankle oedema, from 28 general practices in England.

**Method:**

The outcome was test performance of the CDR and natriuretic peptide test in determining a diagnosis of heart failure. The reference standard was an expert consensus panel of three cardiologists.

**Results:**

Three hundred and four participants were recruited, with 104 (34.2%; 95% confidence interval [CI] = 28.9 to 39.8) having a confirmed diagnosis of heart failure. The CDR+NT-proBNP had a sensitivity of 90.4% (95% CI = 83.0 to 95.3) and specificity 45.5% (95% CI = 38.5 to 52.7). NT-proBNP level alone with a cut-off <400 pg/ml had sensitivity 76.9% (95% CI = 67.6 to 84.6) and specificity 91.5% (95% CI = 86.7 to 95.0). At the lower cut-off of NT-proBNP <125 pg/ml, sensitivity was 94.2% (95% CI = 87.9 to 97.9) and specificity 49.0% (95% CI = 41.9 to 56.1).

**Conclusion:**

At the low threshold of NT-proBNP <125 pg/ml, natriuretic peptide testing alone was better than a validated CDR+NT-proBNP in determining which patients presenting with symptoms went on to have a diagnosis of heart failure. The higher NT-proBNP threshold of 400 pg/ml may mean more than one in five patients with heart failure are not appropriately referred. Guideline natriuretic peptide thresholds may need to be revised.

## INTRODUCTION

Heart failure is a chronic disease associated with significant mortality and poor quality of life.^1^^–^3 Patients may present to primary care with symptoms of gradual-onset breathlessness, fatigue, and ankle swelling.^4^ These symptoms are not unique to heart failure and can be associated with other conditions.^5^^–^7 Making an accurate and timely diagnosis is crucial, and requires referral for objective testing, but deciding who to refer can be challenging.^8^^–^10

Clinical decision rules (CDRs) can help clinicians to assess the probability that a patient has a particular condition.^11^ They are used widely in medicine to inform decisions about investigation and management.^12^^,^^13^ Mant and colleagues developed a CDR for heart failure by undertaking a systematic review that identified 11 prospective studies set in primary care.^14^ The decision rule was derived from an individual patient dataset from one of these studies (Zaphiriou *et al*)^15^ and externally validated on four others that included relevant variables.^16^^–^19

The CDR included three clinical elements, as shown in Box 1, and was combined with N-Terminal pro-B type natriuretic peptide levels (NT-proBNP) to identify those likely to have heart failure and therefore requiring referral for further diagnostic testing.

Box 1. **The ‘MICE’ clinical decision rule**

Refer straight for echocardiography if the patient has any one of:

- a history of myocardial **I**nfarction;
- basal **C**repitations; or
- ankle o**E**dema in a **M**ale.

Otherwise, carry out an NT-proBNP test and refer straight for echocardiography if level is above one of three cut-offs set by sex/symptoms recorded in the clinical rule:

- female without ankle oedema, refer if NT-proBNP >620–1060 pg/ml;
- male without ankle oedema, refer if NT-proBNP >390–660 pg/ml; or
- female with ankle oedema, refer if NT-proBNP >190–520 pg/ml.

Natriuretic peptides are routinely used in the diagnosis of heart failure, although doubt remains about the most appropriate cut-off levels required to optimise diagnostic accuracy.^20^ The European Society of Cardiology (ESC) advocates an NT-proBNP threshold of 125 pg/ml,^8^ below which heart failure can be ruled out, whereas the National Institute for Health and Care Excellence (NICE) in England (where the study took place) recommends a much higher NT-proBNP threshold of 400 pg/ml.^4^

The aim of this diagnostic accuracy study was to assess the performance of the CDR, CDR+NT-proBNP, or NT-proBNP alone in identifying patients with heart failure presenting to primary care.

## METHOD

The full methods for the REFER study have been previously published elsewhere.^21^

**How this fits in**

Patients with symptoms suggestive of heart failure often present to primary care. The diagnosis requires objective evidence of cardiac dysfunction, usually found using echocardiography, but deciding which patients to refer for further testing is challenging. This study found a validated clinical decision rule (CDR) added little to diagnostic accuracy and that N-Terminal pro-B type natriuretic peptide levels (NT-proBNP) testing alone should be carried out in symptomatic patients with suspected heart failure. The cut-off needs to be low enough to ensure cases are not missed.

### Study design and participants

The REFER study was a prospective, observational, diagnostic validation design to assess the performance of the Male, Infarction, Crepitations, Edema (MICE) rule and NT-proBNP level in identifying patients with heart failure. The study population was primary care patients aged >55 years presenting with recent new-onset shortness of breath, lethargy, or peripheral ankle oedema of >48 hours’ duration for which there was no other obvious cause. Patients were excluded if they were unable to give consent, had a previous confirmed diagnosis (that is, with objective evidence) of heart failure, an obvious alternative diagnosis, severe symptoms requiring immediate management, or recent (within 60 days) acute coronary syndrome.

### Recruitment

The original study protocol stated a recruitment target of 500 participants from 20 practices (equivalent to 25 participants per practice) over an 18-month period. Due to difficulties in prospectively recruiting patients within GP appointments at a time of unprecedented demand on the service, the length of the recruitment period was extended and the number of practices increased.

The recruitment phase of the REFER study started on 1 May 2011 and completed on 31 August 2013. Participants were recruited from a random sample of 28 general practices in central England, stratified by practice list size and deprivation quartile.^22^ Participating practices were asked to invite all presenting patients who met the inclusion criteria to join the study consecutively. Assessment was undertaken at the research clinic within 7 days of participants presenting to their GP.

### Assessment clinics

Assessments were carried out within 7 days of recruitment by trained research nurses and an echocardiographer accredited by the British Society of Echocardiography (BSE). Informed consent was obtained, and then detailed clinical history and examination, blood testing, electrocardiograph (ECG), and echocardiogram were carried out. Two attempts at blood taking were allowed. The NT-proBNP level was determined using a point-of-care device (Roche Diagnostics, UK).

### Reference standard

The reference standard was an expert consensus panel of three cardiology specialists, who reviewed each case blinded to the assessments by other panel members. The ESC 2012 guideline was used to define heart failure.^8^ To assess incorporation bias, the panel was presented with clinical information and investigation results in three separate stages. At Step 1, clinical assessment (excluding the CDR variables), ECG, and echo findings were presented. At Step 2, the CDR components (male, history of myocardial infarction, crepitations, and oedema) were added and finally, at Step 3, the NT-proBNP result was included. The cardiology specialists were asked to record if the patient did or did not have heart failure at each of the three steps.

### Statistical methods

A sample of 500 symptomatic patients attending their GP with breathlessness, lethargy, or ankle swelling was proposed. This sample size was sufficient to estimate the sensitivity of the CDR to within 4% and specificity to within 6% at the 95% confidence level. Calculations were based on a sensitivity of 94% and specificity of 48% obtained from the previous individual patient data meta-analysis^19^ and prevalence of heart failure in a symptomatic population of 30%.

Participants with and without a diagnosis of heart failure at Step 3 were compared using independent *t*-tests or Wilcoxon ranked sum tests for continuous measures and χ^2^ tests for categorical variables. The main outcome measures were test performance of the CDR and natriuretic peptide test — alone and in combination — in estimating a diagnosis of heart failure. The findings of the expert consensus panel determined if heart failure — the Observed Disease — was present or absent. The CDR and NT-proBNP results were also used to determine whether heart failure was likely to be present — the Test Disease — and referral for echocardiography would have been indicated. Observed versus Test Disease status was then cross-tabulated to determine the sensitivity and specificity, positive predictive value (PPV), and negative predictive value (NPV) for the CDR, NT-proBNP, and their combination; and also by NT-proBNP cut-offs of 125 pg/ ml and 400 pg/ml suggested by the ESC and NICE guidelines respectively.^4^^,^^8^

The binomial exact method was used to calculate 95% confidence intervals (CIs). Receiver operating characteristics (ROC) curves were generated to determine the overall discriminatory ability of each test in predicting a diagnosis of heart failure. Comparisons were made between performance characteristics of the current cohort and those observed in the original derivation dataset.^15^ The original data used in the analysis are available from the authors.

## RESULTS

### Participants

**Figure 1.**
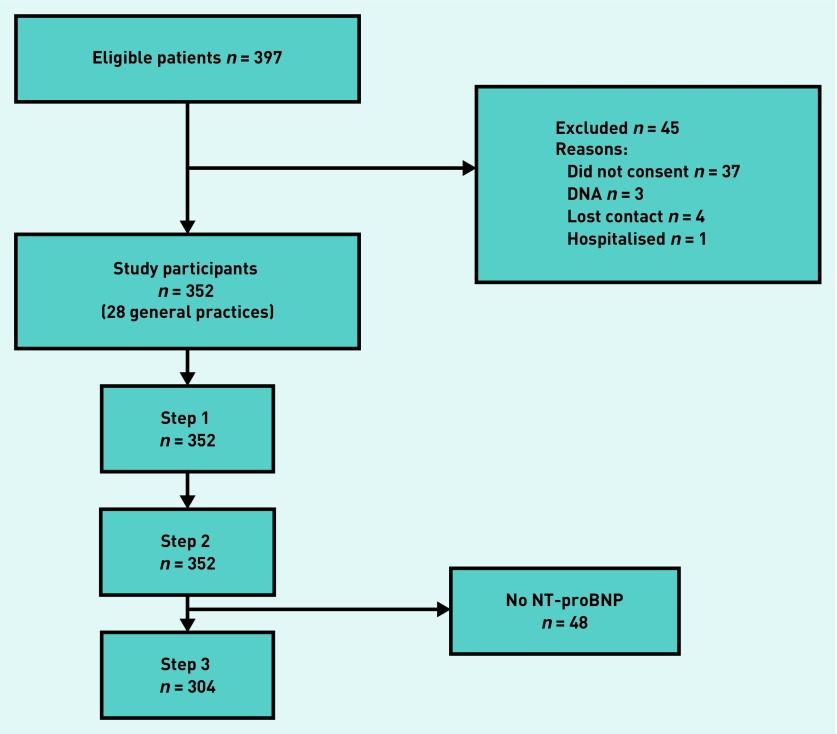
***Flow diagram to show number of participants in REFER study. Step 1 = clinical info +ECG+echo. Step 2 = CDR variables. Step 3 = NT–proBNP result. CDR = clinical decision rule. DNA = did not attend. NT-proBNP = N-Terminal pro-B type natriuretic peptide levels.***

Figure 1 shows a flow diagram for recruitment. Three hundred and ninety-seven patients were eligible for inclusion; 45 were excluded. Of the 352 participants recruited, 48 did not have a blood test (due to failed venepuncture) so were excluded from the final analysis. The remaining 304 participants formed the validation cohort; participants were similar to those excluded, with respect to demography and medical history, except previous record of heart failure, where those without NT-proBNP had a higher prevalence (2.3% versus 8.3%). These heart failure labels from the routine clinical records were, however, not necessarily confirmed with objective evidence or a formal diagnosis.

The clinical and demographic characteristics of the study population are shown in Table 1. The mean age was 73.9 years (standard deviation [SD] 8.8) and 180 (59.2%) participants were female. The cohort had a range of ethnic mix including 18.4% Asian or Asian British. Over half of participants had all three symptoms of breathlessness, ankle oedema, and lethargy. Cardiovascular risk factors such as hypertension and diabetes were prevalent — 221 (72.7%) participants reported having hypertension and 86 (28.3%) had diabetes. Comorbidities were common — 183 participants (60.2%) had arthritis and 73 (24.0%) had depression. Four participants had a record of prior heart failure but this was not confirmed with objective evidence or a formal diagnosis. Cardiovascular medications were commonly prescribed due to the high rate of hypertension in the cohort.

**Table 1.** Participant characteristics of the REFER study and derivation dataset^a^

**Characteristic**	**REFER dataset (*N*= 304)**	**Derivation dataset^15^ (*N*= 298)**	**Comparison, *P*-value^b^**
Age, mean, years (SD)	73.9 (8.8)	71.5 (11.5)	0.004

Male	124 (40.8)	122 (40.9)	0.9700

Ethnicity			
White	214 (70.4)	–	–
Asian/Asian British	56 (18.4)	–	–
Black/black British	16 (5.3)	–	–
Other	18 (5.9)	–	–

Ankle oedema	248 (81.6)	191 (64.1)	<0.0001

Breathlessness	247 (81.3)	283 (95.0)	<0.0001

Lethargy	226 (74.3)	184 (62.1)	0.0009

Previous myocardial infarction	34 (11.2)	42 (14.1)	0.2800

Basal crepitations	16 (5.3)	81 (27.2)	<0.0001

Hypertension	221 (72.7)	165 (55.4)	<0.0001

Diabetes	86 (28.3)	57 (19.1)	0.0083

COPD	17 (5.6)	57 (19.1)	<0.0001

Depression	73 (24.0)	–	–

Arthritis	183 (60.2)	–	–

Medications			
ACE inhibitors	98 (32.2)	68 (22.8)	0.0100
Beta-blockers	82 (27.0)	68 (22.8)	0.2400
ARBs	58 (19.1)	–	–
Diuretics	136 (44.7)	189 (63.4)	<0.0001

NT-proBNP median [IQR]	214 [79–494]	381.5 [135–1187]	<0.0001

a Figures are N (%) unless stated otherwise.

b Means of the two datasets were compared using a two-sample t-test; medians with a Wilcoxon ranked sum test; and proportions with χ^2^ tests. ACE = angiotensin-converting enzyme. ARBs = angiotensin receptor blockers. COPD = chronic obstructive pulmonary disease. IQR = interquartile range. SD = standard deviation. NT-proBNP = N-Terminal pro-B type natriuretic peptide levels.

The REFER cohort, although similar in age and sex to the derivation dataset,^15^ had fewer referrals due to shortness of breath and more due to ankle oedema and lethargy. Hypertension and diabetes were observed in greater frequency in the REFER population but a lower proportion of patients had chronic obstructive pulmonary disease (COPD). Prescribing of diuretics was less frequent in the REFER cohort but a higher proportion were prescribed angiotensin-converting enzyme (ACE) inhibitors.

### Number of participants with heart failure

The expert panel reviewed the data for each participant and determined whether or not a heart failure diagnosis was present; 104 participants had heart failure, which represented 34.2% (95% CI = 28.9 to 39.8) of the cohort. The objective abnormalities found on ECG and echo are shown in Table 2.

**Table 2.** Objective abnormalities found on ECG and echo in participants with and without heart failure

**Abnormality^a^**	**Heart failure *N* (%)**	**No heart failure *N* (%)**
Moderate to severe LVSD — ejection fraction ≤40%	3 (2.9)	0 (0)
Borderline LVSD — ejection fraction 41–50%	9 (8.7)	1 (0.5)
Diastolic dysfunction	15 (14.4)	6 (3.0)
Significant valve disease	47 (45.2)	17 (8.5)
Atrial fibrillation	33 (31.7)	0 (0)
All	104	200

a Some participants had >1 abnormality. ECG = electrocardiogram. LVSD = left ventricular systolic dysfunction.

The characteristics of participants with and without heart failure are shown in Table 3. Participants with heart failure were older and half were male. Presenting symptom profile was similar. Proportionately more patients with heart failure had a history of myocardial infarction (16.4% versus 8.5%) but there was no significant difference in other comorbidities such as hypertension, COPD, and arthritis. Depression was more common in the non-heart-failure group. Cardiovascular medications were more likely to be prescribed in those with heart failure than those without heart failure.

**Table 3.** Characteristics of REFER participants with and without heart failure

**Characteristic**	**Heart failure (*N*= 104) *N* (%)**	**No heart failure (*N*= 200) *N* (%)**	**Heart failure versus no heart failure *P*-value**
Age, years, mean (SD)	77.4 (7.4)	72.1 (9.0)	<0.0001
Male	52 (50.0)	72 (36.0)	0.0200
BMI, kg/m^2^	29.1 (5.7)	31.1 (6.7)	0.0080
Breathlessness	84 (80.8)	163 (81.5)	0.8800
Ankle oedema	87 (83.7)	161 (80.5)	0.5000
Lethargy	72 (69.2)	154 (77.0)	0.1400
Basal crepitations	4 (3.9)	12 (6.0)	0.4200
Previous myocardial infarction	17 (16.4)	17 (8.5)	0.0400
Hypertension	79 (76.0)	142 (71.0)	0.3600
Diabetes	29 (27.9)	57 (28.5)	0.9100
Depression	17 (16.4)	56 (28)	0.0200
COPD	7 (6.7)	10 (5)	0.5300
Arthritis	55 (52.9)	128 (64)	0.0600
ACE inhibitors	38 (36.5)	60 (30.0)	0.2500
Beta-blockers	46 (44.2)	36 (18.0)	<0.0001
ARBs	19 (18.3)	39 (19.5)	0.8000
Diuretics	61(58.6)	75 (37.5)	0.0004
NT-proBNP (pg/ml) median [IQR]	715.5 [413 to 1559]	126 [60 to 233]	<0.0001
NT-proBNP ≥ 125 pg/ml *N* (%)	98 (94.2)	102 (51.0)	<0.0001
NT-proBNP ≥ 400 pg/ml *N* (%)	80 (76.9)	17 (8.5)	<0.0001

ACE = angiotensin-converting enzyme. ARBs = angiotensin receptor blockers. BMI = body mass index. COPD = chronic obstructive pulmonary disease. IQR = interquartile range. SD = standard deviation. NT-proBNP = N-Terminal pro-B type natriuretic peptide levels.

The median NT-proBNP level was significantly higher in the heart failure group. At the lower 125 pg/ml cut-off, over half of patients without heart failure had an NT-proBNP above the threshold for referral to echocardiography.

### Diagnostic accuracy estimates

The diagnostic accuracy of the CDR, NT-proBNP level, and their combination is shown in Table 4. The clinical information (MICE symptoms) of the CDR had a sensitivity of 44.2% (95% CI = 34.5 to 54.3), but with the addition of the NT-proBNP level at the lower cut-offs this improved to a sensitivity of 90.4% (95% CI = 83.0 to 95.3) and specificity 45.5% (95% CI = 38.5 to 52.7). NT-proBNP level alone with a cut-off less than 400 pg/ml had sensitivity 76.9% (95% CI = 67.6 to 84.6) and specificity 91.5% (95% CI = 86.7 to 95.0). At the lower cut-off of 125 pg/ml, sensitivity was 94.2% (95% CI = 87.9 to 97.9) and specificity 49.0% (95% CI = 41.9 to 56.1).

**Table 4.** Performance characteristics of the clinical decision rule and NT-proBNP

	**AUROC**	**Sensitivity 95% CI**	**Specificity 95% CI**	**PPV 95% CI**	**NPV 95% CI**
**Derivation dataset^15^**					
CDR (lower cutoffs)^a^	0.74 (0.70 to 0.79)	90.2 (82.7 to 95.2)	58.2 (50.9 to 65.2)	52.9 (42.6 to 64.8)	91.9 (75.8 to 100)
CDR (upper cutoffs)^b^	0.75 (0.70 to 0.80)	87.3 (79.2 to 93.0)	62.2 (55.5 to 69.1)	54.6 (46.6 to 62.4)	90.4 (84.1 to 94.8)

**REFER**					
CDR (MICE variables)	0.54 (0.48 to 0.60)	44.2 (34.5 to 54.3)	64.0 (56.9 to 70.6)	39.0 (30.1 to 48.4)	68.8 (61.6 to 75.4)
CDR+NT-proBNP (lower cut-offs^a^)	0.68 (0.64 to 0.72)	90.4 (83.0 to 95.3)	45.5 (38.5 to 52.7)	46.3 (39.3 to 53.4)	90.1 (82.5 to 95.1)
CDR+NT-proBNP (upper cutoffs^b^)	0.71 (0.66 to 0.76)	78.8 (69.7 to 86.2)	63.5 (56.4 to 70.2)	52.9 (44.7 to 61.0)	85.2 (78.5 to 90.5)
NTproBNP≥125pg/ml alone	0.72 (0.67 to 0.76)	94.2 (87.9 to 97.9)	49.0 (41.9 to 56.1)	49.0 (41.9 to 56.1)	94.2 (87.9 to 97.9)
NT-proBNP≥400pg/ ml alone	0.84 (0.80 to 0.89)	76.9 (67.6 to 84.6)	91.5 (86.7 to 95.0)	82.5 (73.4 to 89.4)	88.4 (83.2 to 92.4)

a Post-test probability of 20%.

b *Post-test probability of 30%. AUROC = area under the receiver operating curve. CDR = clinical decision rule. MICE = Male, Infarction, Crepitations, Edema. NPV* = *negative predictive factor. PPV* = *positive predictive factor. NT-proBNP = N-Terminal pro-B type natriuretic peptide levels.*

**Figure 2.**
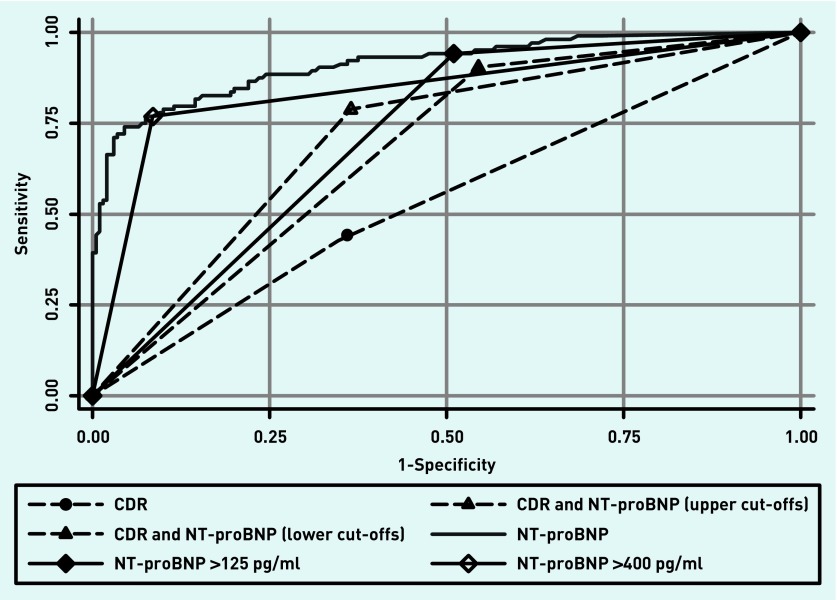
***Receiver operating characteristic curves of CDR+NT-proBNP or NT-proBNP alone for predicting heart failure. CDR = clinical decision rule. NT-proBNP = N-Terminal pro-B type natriuretic peptide levels.***

These performance characteristics were mostly lower in magnitude than the corresponding values observed in the derivation dataset. However, comparison of the CIs suggests that the differences were not statistically different at the 5% level. Figure 2 shows the ROC curves of each index test for predicting heart failure. Significant differences (*P*<0.0001) were observed between the areas under the receiver operating curves (AUROCs) shown in Table 4. NT-proBNP had the best discriminatory power with AUROC of 0.91 (95% CI = 0.88 to 0.95) and the clinical element (MICE) of the CDR the poorest with AUROC 0.54 (95% CI = 0.48 to 0.60).

### Assessing incorporation bias

The performance characteristics for the CDR+NT-proBNP or NT-proBNP alone at Steps 1 to 3 are shown in Appendix 1. The diagnostic accuracy of all tests increased at each step, with largest changes observed when NT-proBNP was used without the clinical element of the CDR. NT-proBNP cut-off 400 pg/ml showed a statistically significant increase in the detection of cases without heart failure from Step 2 to Step 3 (*P*<0.05).

## DISCUSSION

### Summary

The CDR was not clinically helpful in isolation; NT-proBNP testing alone performed as well as the validated CDR in determining which patients presenting with possible heart failure symptoms went on to have a diagnosis of heart failure. At a NT-proBNP threshold of 125 pg/ml, as advised in ESC guidance, 94% of patients who went on to have heart failure were identified. However, at an NT-proBNP threshold of 400 pg/ml, the current level recommended by NICE in England, only 77% of heart failure patients were appropriately referred. More than one in five patients in this study would have been misdiagnosed.^4^

### Strengths and limitations

This study included patients presenting prospectively to their GP. A large proportion of health care in England is provided through general practice, and testing the CDR in a real-life clinical setting where most patients are managed allows accurate validation of the rule.^23^ Participants underwent thorough phenotyping, including clinical and objective assessment. The data were then reviewed by a panel of three experienced cardiologists, using a staged system to allow for assessment of incorporation bias, to agree a formal diagnosis so that the ‘Observed Disease’ was accurate.^24^ The study was slow to recruit and failed to meet the initial target of 500 patients. This was due to the requirement to recruit within the routine 10-minute consultation at a time of unparalleled increased workloads in English general practice.^25^ Furthermore, when the study was designed, natriuretic peptides were not routinely available and therefore the provision of natriuretic peptide testing and a rapid diagnostic service, via the REFER study, might have been attractive to GPs. However, shortly after the study commenced, natriuretic peptide assays became an open-access diagnostic for practices in the region.^4^ Although fewer participants were recruited than planned, the performance characteristics of the MICE rule were estimated with only marginally lower precision than designed. These findings also represent the largest diagnostic accuracy study conducted in patients with undifferentiated symptoms presenting to primary care with possible heart failure.

The number of participants with heart failure due to reduced ejection fraction was unexpectedly low in the cohort. This may reflect the nature of heart failure presentation where those with left ventricular systolic dysfunction may be more likely to present acutely direct to secondary care, or may already be under the care of a cardiologist for a known cardiovascular comorbidity such as coronary artery disease.^26^

The increase in performance across the stepped diagnosis suggests that the sensitivity of the index tests may have been overestimated due to incorporation bias. However, evaluation of the results at Step 2 (where NT-proBNP was excluded from clinical diagnosis) confirms that the diagnostic accuracy of the NT-proBNP test alone at the lower cut-off is similar to that of the CDR+NT-proBNP.

### Comparison with existing literature

Heart failure can be a difficult diagnosis to make and the idea of a CDR to help primary care clinicians with the decision of who to refer for objective testing is justifiable.^27^ The role of CDRs as an aid to clinical decision-making, however, remains controversial. There are many examples of CDRs being generated and validated with the hope of improving clinical accuracy but performance characteristics are often modest at best.^28^^–^30 Furthermore, remembering the components of a CDR and applying it within the consultation can be challenging for busy generalist clinicians seeing patients with undifferentiated illness.^31^

The reason the CDR performed no better than NT-proBNP alone may be due to the diagnosis of heart failure in the cohort being largely heart failure with preserved ejection fraction. This spectrum of patients was different from previous studies of heart failure where the prevalence of a low ejection fraction (<40%) was more common. The derivation and initial validation of the CDR relied predominantly on epidemiological studies, which included heart failure with reduced ejection fraction, so may not directly apply to the REFER population.^14^ In addition, the way symptoms were recorded may have differed: the study that was used to derive the CDR was carried out by cardiologists in a secondary care clinic, whereas the REFER study data were collected by research nurses. However, both studies relied on referral from primary care. The prevalence of atrial fibrillation and valvular disease was also very high in the REFER cohort. This may reflect a new reality where clinically florid cases of heart failure with reduced ejection fraction present to acute services, while primary care experiences an increase in the number of patients with heart failure with preserved ejection fraction, and/or other cardiovascular comorbidities.^32^

### Implications for research and practice

The threshold for NT-proBNP below which heart failure can be reasonably excluded is also an area of ongoing research.^33^ ^34^ Cost effectiveness is an important consideration at a population level and is being carried out using the results of the REFER study. For any test, there is always a trade-off between sensitivity and specificity,^28^ and guidelines differ in the threshold they currently recommend.^4^^,^^8^ A high sensitivity ensures fewer cases are missed, but at the expense of more patients undergoing echocardiography, a test with limited availability in many healthcare systems including the NHS in England. But accepting a test with a sensitivity that is too low could result in a diagnosis of heart failure being missed. This study shows that, in patients suspected of having heart failure, an NT-proBNP blood test alone, at a threshold of 125 pg/ml, means heart failure is unlikely and thus could be used as a ‘rule out’ test to reduce the burden on echo services. At the higher NT-proBNP threshold of 400 pg/ml more than one in five cases of heart failure may be missed. Guidelines should be revised to ensure natriuretic peptide cut-off levels are low enough to ensure GPs are not falsely reassured that referral for echocardiography is not required.

## Appendix. Performance characteristics of the CDR and NT-proBNP at Steps 1 to 3

**Table:**

	**Sensitivity (95% CI)**	**Specificity (95% CI)**	**PPV (95% CI)**	**NPV (95% CI)**
**CDR+NT-proBNP (lower cut-offs)**				
Step 1	78.8 (67.0 to 87.9)	36.6 (30.4 to 43.0)	25.6 (19.8 to 32.2)	86.1 (77.8 to 92.2)
Step 2	85.4 (76.3 to 92.0)	40.9 (34.3 to 47.8)	37.4 (30.8 to 44.5)	87.1 (79.0 to 93.0)
Step 3	90.4 (83.0 to 95.3)	45.5 (38.5 to 52.7)	46.3 (39.3 to 53.4)	90.1 (82.5 to 95.1)

**CDR+NT-proBNP (upper cut-offs)**				
Step 1	60.6 (47.8 to 72.4)	51.7 (45.1 to 58.2)	25.8 (19.1 to 33.4)	82.6 (75.5 to 88.3)
Step 2	74.2 (63.8 to 89.2)	58.6 (51.7 to 65.3)	42.6 (34.7 to 50.8)	84.6 (77.7 to 90.0)
Step 3	78.8 (69.7 to 86.2)	63.5 (56.4 to 70.2)	52.9 (44.7 to 61.0)	85.2 (78.5 to 90.5)

**NT-proBNP** >**125**				
Step 1	81.8 (70.4 to 90.2)	38.7 (32.4 to 45.2)	27.0 (21.0 to 33.7)	88.5 (80.7 to 93.9)
Step 2	84.3 (75.0 to 91.1)	41.9 (35.2 to 48.8)	37.5 (30.8 to 44.6)	86.5 (78.4 to 92.4)
Step 3	94.2 (87.9 to 97.9)	49.0 (41.9 to 56.1)	49.0 (41.9 to 56.1)	94.2 (87.9 to 97.9)

**NT-proBNP** >**400 pg/ml**				
Step 1	48.5 (36.0 to 61.1)	72.7 (66.6 to 78.2)	33.0 (23.8 to 43.3)	83.6 (77.8 to 88.3)
Step 2	58.4 (47.5 to 68.8)	79.1 (73.0 to 84.3)	53.6 (43.2 to 63.8)	82.1 (76.2 to 87.1)
Step 3	76.9 (67.6 to 84.6)	91.5 (86.7 to 95.0)	82.5 (73.4 to 89.4)	88.4 (83.2 to 92.4)

*CDR* = *clinical decision rule. NPV* = *negative predictive factor. PPV* = *positive predictive factor. NT-proBNP = N-Terminal pro-B type natriuretic peptide levels.*
